# Pharmacy students’ insights on assessment tools and their predictive value in evaluating clinical competencies

**DOI:** 10.1186/s12909-026-09273-w

**Published:** 2026-04-23

**Authors:** Adeladlew Kassie Netere, Ashenafi Kibret Sendekie, Gashaw Sisay Chanie, Eyayaw Ashete Belachew

**Affiliations:** School of Pharmacy, Department of Clinical Pharmacy, University of Gondar, Gondar, Ethiopia

**Keywords:** Assessment, Clinical competence, Competency-based education, Pharmacy, Students, Perception

## Abstract

**Introduction:**

Competency-based pharmacy education (CBPE) emphasizes patient-centred care and clinical accountability, requiring the translation of core competencies into measurable learning outcomes supported by effective assessment strategies. Within this framework, both formative and summative assessments are essential. However, evaluating clinical competence remains challenging due to the limitations of current methods. Understanding of students’ perspectives is therefore crucial for refining these strategies. This study explores pharmacy students’ perceptions of assessment tools and their perceived effectiveness in evaluating clinical competencies at the University of Gondar.

**Method:**

A cross-sectional survey was conducted among graduating pharmacy clerkship students at the College of Medicine and Health Sciences, University of Gondar, Ethiopia, who had completed a one-year clinical attachment across multiple medical wards. Data were collected in August 2022 using a structured, self-administered face-to-face questionnaire. The survey explored students’ perceptions of assessment instruments and approaches used within the competency-based pharmacy curriculum. Descriptive statistics and comparative analyses were conducted across multiple assessment instruments to evaluate perceived difficulty, fairness, degree of learning, and frequency of use, using the Friedman test.

**Result:**

Of the 135 students approached, 118 participated (response rate 87%). Approximately 64% were regular fifth-year undergraduate pharmacy students in the clerkship program. The formative assessment approach was perceived to enhance confidence (68%) and motivation to study (64%). In the summative assessment domain, 52% of students agreed or strongly agreed that continuous assessment is a fairer method for evaluating academic performance compared to one-off exams. About 50% of students rely primarily on question-spotting as their strategy for preparing for written exams. Furthermore, 47% and 46% agreed and strongly agreed that module grades often depend too heavily on written exams, and that study materials were excessively demanding, respectively. Also, many students (68%) agreed on the utility and objectivity of the Objective Structured Clinical Examination (OSCE) in assessing clinical competencies; they rated it as difficult to perform.

**Conclusion:**

This survey showed that students view formative assessments as helpful for enhancing learning, motivation, and confidence. In contrast, summative assessments mainly decide grades through a single examination. Although many students found OSCE stations challenging, these tools were considered effective for evaluating clinical skills. These results offer valuable insights into students’ experiences with different assessment methods and can inform the selection and improvement of assessment strategies in competency-based pharmacy education.

## Introduction

### Clinical competency

In the healthcare system, competency-based educational models are becoming dominant, appealing to both national and international interests [1, 2]. The growing demand for pharmacists to excel in patient care and to prioritize advanced healthcare and patient safety is the primary driver of this shift [3, 4]. Pharmacy education in many countries has progressively shifted from a technical, product-oriented focus towards patient-centred professional services, although the extent of this transformation varies globally. Within this evolving model of practice, pharmacists are expected to assume preprofessional responsibility and accountability for optimizing medication therapy and contributing to improved patient outcomes [5–7].

However, executing competency-based pharmacy education (CBPE) is a complex and challenging task that requires thoughtful translation of defined competencies into specific learning outcomes and assessment methods [8]. Outcome-based pharmacy education employs a competency-based assessment approach endorsed by national and international administrative bodies, which is progressively producing skilled pharmacists who can effectively meet health needs. Ensuring the required quality is essential for certifying graduates’ qualifications, yet challenging without a competency-based approach [9]. Competency-based education is now key to training healthcare professionals, including pharmacists, and calls for assessment models that effectively evaluate clinical competency at various learning stages. Miller’s pyramid offers a well-founded framework that covers knowledge (knows), application (knows how), demonstration (shows how), and actual performance (does) [10]. Several competency frameworks also shape the development and evaluation of pharmacy education outcomes. For instance, the Centre for the Advancement of Pharmacy Education (CAPE) provides a specified approach to learning outcomes focused on knowledge, skills, and cognitive requirements necessary for entry-level pharmacists in the USA. These learning goals provide a structured framework for assessing pharmacy degree graduates, encompassing core elements compulsory for safe and appropriate drug therapy [11, 12]. Internationally, the International Pharmaceutical Federation (FIP) Global Competency Framework provides a globally relevant structure for defining pharmacist competencies and supporting workforce development across different practice environments [13].

The competency standards for pharmacy graduates rely heavily on diverse assessment methods and assessors embedded in effective educational systems, through which a variety of assessment tools are used [1]. The tools can ensure the required quality levels and competencies in graduates, though careful design is needed to avoid misinterpretation when integrating them into daily pharmacy practices [14, 15]. Multiple assessment methods are crucial for achieving competency-based medical education (CBME). These include written exams, OSCEs, workplace assessments, observation of procedural skills, and portfolio and reflective assessments. Within these methods, formative feedback is vital for supporting ongoing competency development [5, 16]. Although pharmacy education in many settings has gradually shifted towards a competency-based approach, evaluating competencies remains challenging in the field, and designing effective assessment strategies continues to evolve across various educational environments [17, 18]. This shift has highlighted the limitations of current assessment methods [18] and necessitates strategies to more effectively assess competencies. Therefore, inclusive global cooperation in research and practice is needed to ensure the validity and authenticity of the competence-based assessment methods [17].

### Assessment tools of clinical competency

Assessment in competency-based education (CBE) is a cooperative and ongoing approach that collects data to improve teaching and learning outcomes [19]. It evaluates learners’ capabilities to provide safe and clinically competent patient care [20]. The main goal is to encourage learning and assess preparedness for advancement [21]. Effective CBE assessments require dedicated efforts to provide continuous feedback and depend on trained evaluators [5]. Presently, there is no agreement on assessment tools to gauge students’ competencies, rendering a universal approach unfeasible [22]. Therefore, it is crucial to incorporate existing assessment frameworks to mitigate the weaknesses of individual methods [23]. A well-rounded evaluation of practical applications should integrate both assessment for learning (AFL) and assessment of learning (AOL) principles [24]. To enhance the efficacy of these assessment models, it is important to consider students’ perspectives and attitudes, as these can improve the predictive relevance of these models in clinical competency assessment.

In the educational assessment process, assessment for learning (AFL) and Assessment of Learning (AOL) are two complementary yet distinct approaches. AFL is a continuous process that provides instant, actionable feedback to both students and teachers throughout instruction. This approach is designed to inform teaching strategies and support student learning through ongoing, formative assessments (FA) such as quizzes, case presentations and interactive activities. By engaging students in self-assessment and reflection, AFL aims to identify areas for growth and improvement, fostering a more adaptive and responsive educational environment [25–27].

On the other hand, AOL aims to assess and rectify student learning at the end of an instructional period and measure the extent of student learning against predefined standards. This summative assessment encompasses final exams, Objective Structured Clinical Examination (OSCE), Oral exams and end-of-term projects. This approach is high-stakes, used for grading, certification, and accountability purposes, providing a cumulative assessment of what students have learned and can demonstrate [28].

While AOL serves as a final measure of educational outcomes, AFL directly influences teaching and learning processes by offering continuous feedback and opportunities for improvement [29]. Assessment of clinical competency models is tailored to performance levels, learning stages, academic organization capabilities, and whether the purpose is formative or summative [5, 10]. Since 2013, Ethiopia has implemented a modularized competency-based curriculum for pharmacy students, organizing courses into integrated modules aligned with specific competency goals [30]. The assessment for the bachelor’s degree in pharmacy, outlined by the *Nationally Harmonized Modular Curriculum*,* involve*s both formative and summative methods. Formative assessments encompass ongoing evaluations such as tests, quizzes, case presentations, assignments, bedside rounds, supervision, logbooks, and practical skill assessments. Summative assessments include final written exams, OSCEs, oral exams, and both internal and external comprehensive exams (Curriculum, 2013, p. 12) [30]. This study seeks to explore pharmacy students’ perspectives on these assessment tools and examine how well clinical competencies predict future performance among clinical pharmacy students at the University of Gondar. These assessment strategies offer complementary advantages in evaluating clinical competence. Formative assessments enable ongoing learning and deliver timely feedback to foster skill growth, whereas summative assessments provide standardized benchmarks of competency [16, 26, 31]. Nevertheless, using multiple assessment methods can pose challenges, including a greater faculty workload, the need for trained evaluators, and the need to maintain reliability and consistency across different assessment environments.

## Method and materials

### Study design and instruments

A cross-sectional survey was conducted to examine students’ attitudes and insights towards assessment approaches in pharmacy education, with a focus on their current and future learning, as well as the predictive value of available assessment methods used in Ethiopian clerkship attachments and their correlation with clinical competency. It employs a comparative descriptive research design and aims to describe the assessment practices adopted by pharmacy educators in Ethiopia to evaluate clinical skills or competence.

### Assessment and evaluation mechanisms

The Bachelor of Pharmacy program uses a nationally standardized, modular, competence-based curriculum. Courses are arranged into integrated modules designed to develop specific professional skills. The five-year course combines classroom learning, laboratory work, and clinical placements, including hospital and community clerkships. Student performance is evaluated through a combination of ongoing (formative) and final (summative) assessments. Ongoing assessments include tests, quizzes, case presentations, assignments, bedside rounds, supervision, logbooks, and practical skill evaluations. Summative assessments consist of final written exams, OSCEs, oral exams, and both internal and external comprehensive assessments to evaluate competency [30].

### Study institution and participants

This research was conducted at the College of Medicine and Health Sciences, University of Gondar, Ethiopia, focusing on pharmacy students who completed a one-year clinical clerkship in various clinical wards. In Ethiopia, the Bachelor of Pharmacy degree is available through two pathways: the traditional five-year undergraduate program and a post-basic program designed for students with previous pharmacy qualifications, typically lasting three to four years. Despite the variation in duration, students from both tracks complete the same one-year clinical clerkship in their final year. Only students who completed this clerkship were included, as they experienced all assessment methods within the clinical pharmacy curriculum and could provide feedback on these approaches. Data collection was conducted in August 2022, when students completed their clerkship program and were presumed to be qualified in clinical competencies. Data were collected through a self-administered, face-to-face survey, and all eligible students were invited to participate. As convenience sampling was employed to include the entire accessible group, no sample size calculation was necessary. The survey took approximately 30 to 60 min to complete.

### Data collection instrument

A self-administered questionnaire was used to gather data. The questionnaire was developed and tailored to our setting, based on the hierarchical framework of clinical competence described by Mehay and Burns [33] in the Revisiting Miller’s pyramid model [32] for medical education assessments. This model improves upon Miller’s original concept by arranging clinical competence into four levels: Knows, Knows How, Shows How, and Does [10, 32]. Each level corresponds with specific assessment methods used in the curriculum, in which evaluation is conducted through both formative assessment (FA) and summative assessment (SA).

The “Knows” level, which involves knowledge acquisition, is primarily assessed using summative methods such as final written exams and internal/external comprehensive exams. The “Knows How” level, which reflects the application of knowledge, is evaluated through a combination of formative assessments, including tests, quizzes, case presentations, and assignments, as well as summative assessments such as oral exams. The “Shows How” stage, which focuses on demonstrating clinical skills in simulated environments, is assessed using OSCEs (summative) and practical skill evaluations (formative). The “Does” level, representing real-world performance, is assessed through workplace-based formative assessments, including bedside teaching, clinical supervision, and logbook documentation.

Furthermore, the OSCE assessment-related items were adapted from previous publications with some modifications to the study settings [33, 34]. The questionnaire also included aspects of integrative assessment practices, highlighting the complementary roles of current and future learning. It emphasizes both formative and summative assessments to promote ongoing learning and competency development, as well as to support self-regulated and long-term learning practices [31, 35, 36]. This integrated framework allows for a comprehensive evaluation of students’ perceptions of assessment methods across all stages of the updated Miller’s clinical competence pyramid.

Data collection items were created to align with each level of this framework in the context of the assessment mechanisms used in the Nationally Harmonized Curriculum [30]. Although no standardized original questionnaire was available, the items were carefully developed and reviewed by pharmacy educators to ensure content validity and contextual relevance. Items were generated to reflect the activities and assessment tools used in the curriculum and were mapped against the four levels of Miller’s pyramid. Two other pharmacy education experts assessed the clarity and relevance before final administration.

The questionnaire comprises four sections: (1) participants background information and characteristics; (2) items (*n* = 12) exploring opinions and attitudes on formative and summative assessments methods; (3) items (*n* = 17) assessing understanding and views on the OSCE assessment methods; and (4) questions exploring different clinical assessment formats and rating of their difficulty, fairness, degree of learning, and frequency of application in assessing clinical competencies. This comparison section included closed-ended questions. Responses to assessment methods were measured on a five-point Likert scale (where 1 = strongly disagree, 2 = disagree, 3 = uncertain, 4 = agree, and 5 = strongly agree) to assess responses to assessment methods, and a three-point scale for evaluating the difficulty, fairness and preference of OSCE compared to other assessment methods. The direction of Likert scale scoring varied depending on the construct being measured. For perceived difficulty, higher scores indicate greater ease; for fairness, degree of learning, and preferred frequency of use, higher scores reflect more positive responses (e.g., greater fairness, greater learning, or stronger preference).

### Statistical analysis

After completing the paper-based questionnaire, quantitative data were coded and entered into a customized database for statistical analyses using IBM SPSS Statistics for Windows, Version 28.0. (Armonk, NY). Descriptive statistics were primarily used to present the participants’ socio-demographic characteristics. Frequency analysis was performed to present differences in respondents’ perceptions of assessment methods. Tables and graphs were utilized to display findings. A comparative measure was conducted across multiple assessment instruments to evaluate perceived difficulty, fairness, degree of learning and preferred frequency of use, using the Friedman test.

## Results

### Sociodemographic characteristics of the study participants

Of the 135 students approached, 118 participated in this study, resulting in a response rate of 87%. The participants were mainly male (*n* = 63, 53%) with an average age of 26 (± 3). Most respondents (*n* = 75, 64%) were regular undergraduate clinical pharmacy students enrolled in the five-year program. The remaining participants (*n* = 43, 36%) were post-basic students with prior pharmacy qualifications who followed a three to four-year training pathway. Despite their different pathways, all participants completed the same one-year clinical clerkship training, which was a requirement for inclusion in this study.

### Students’ attitudes towards the formative and summative assessment approach

**Fig. 1:**
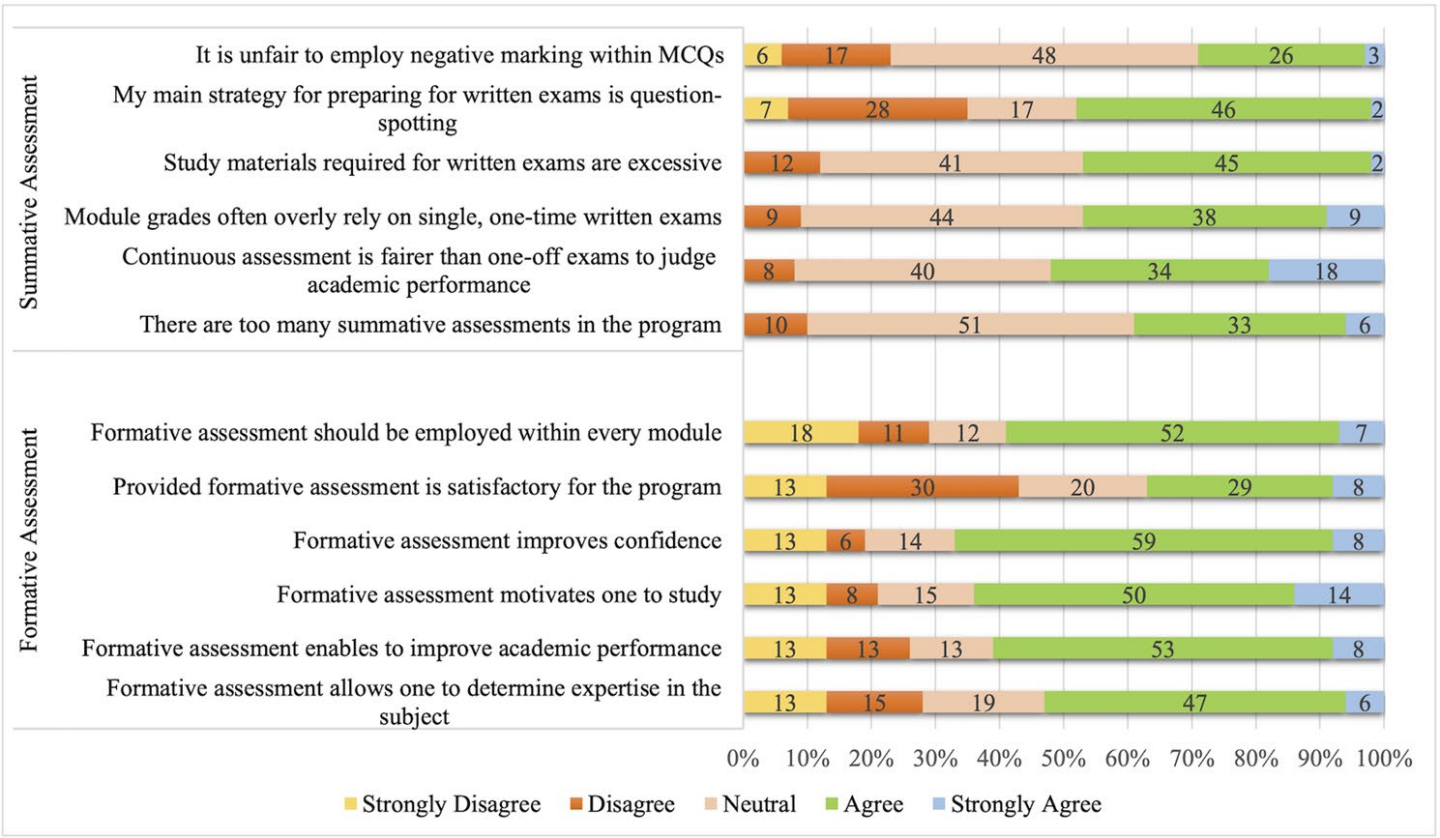
Students’ attitudes towards formative and summative assessment approaches

Overall, students demonstrated positive attitudes toward the formative assessment (FA) approach. Across items, 29–59% of students selected “agree,” and 6–14% selected “strongly agree.” Median scores ranged from 3 to 4 on the 5-point Likert scale, with interquartile ranges (IQR) of 2–4 to 3–4, indicating that responses generally clustered around the “agree” category. About two-thirds of the respondents agreed or strongly agreed that formative assessment (FA) increased motivation to study (64%), improved academic performance (62%), and boosted confidence (67%). Regarding summative assessment, median item scores ranged from 3 to 4 (IQR: 2–4 to 3–4) on the 5-point Likert scale. More than half of the students (52%) believed that continuous assessment is fairer than single examinations for assessing academic performance. Around half of the students (48%) reported question-spotting as their main strategy for preparing for written exams. Additionally, 47% agreed or strongly agreed that the study materials for written exams were excessive, while 47% felt that module grades rely too much on single, one-off written assessments (Fig. 1).

### Participants’ attitudes towards the OSCE assessment method

**Fig. 2:**
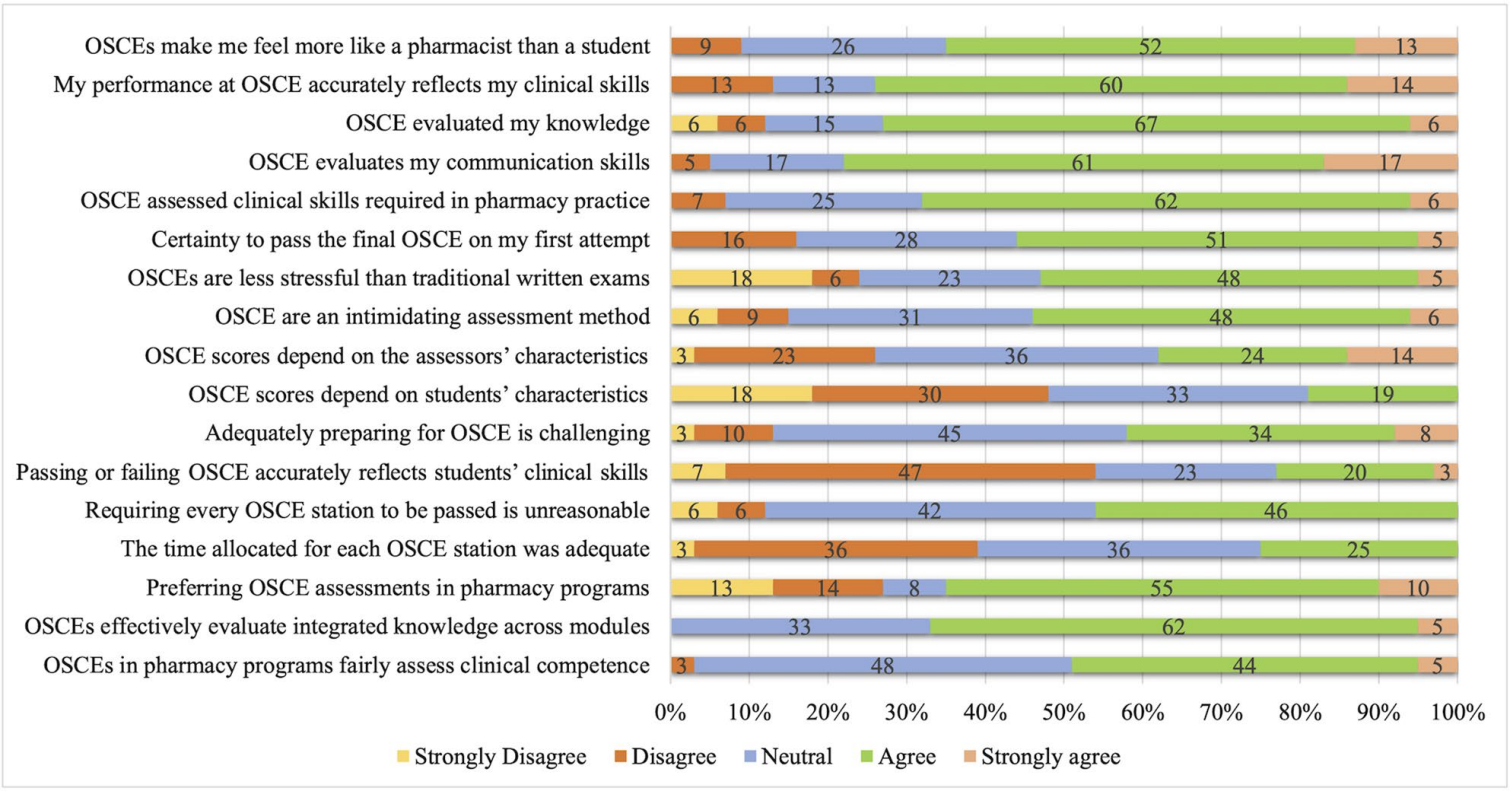
Students’ attitudes towards OSCE assessments

Overall, students showed generally positive attitudes towards the OSCE assessment method. Across most items, 48–67% of students selected “agree,” and 5–17% selected “strongly agree,” with median scores ranging from 3 to 4 (IQR: 3–4) on the 5-point Likert scale. About 73% of participants agreed or strongly agreed that OSCE effectively evaluated their knowledge. Similarly, around 78% reported that OSCE effectively assesses communication skills, and 74% agreed or strongly agreed that OSCE measures clinical skills needed in pharmacy practice (Fig. 2).

### Students’ rating of assessment instruments

Students were asked to review the competency assessment tools for their difficulty, fairness, and effectiveness in promoting learning, as well as their preferences regarding how often these tools should be used. A substantial number of students either refrained from expressing opinions or took ambiguous positions regarding the difficulty, fairness, and usage preferences of the assessment instruments. Approximately half (50%) of the students found essay-type questions the hardest, followed by the OSCE (33%). In contrast, portfolios (58%) and multiple-choice questions (MCQs) (47%) were seen as the simplest assessment methods. In terms of fairness, 52% of students viewed MCQsS as the most equitable, closely followed by seminar or journal-based presentations at 47%, while 20% regarded the oral exam as unfair. When assessing the effectiveness of knowledge assessment tools, about 60% of students reported that significant learning could be achieved through both MCQs and OSCEs. Moreover, a notable number of students believe these methods should be incorporated more often into the curriculum, with 48% and 47% respectively, highlighting their perceived educational benefit. Conversely, only a small proportion of students reported learning from oral exams (20%) and easy questions (18%), citing infrequent use of the latter. The Friedman test showed a significant difference in the perceived difficulty of the assessment instruments, χ² [6] = 219; *p* < 0.001. In this analysis, higher scores indicate a more difficult perception of difficulty. For instance, portfolios were most often rated as easy (58%), while easy questions were most deemed difficult (50%). Students also reported a significant difference in their perceptions of the fairness of the assessment tools used to evaluate clinical competence, χ² [6] = 83; *p* < 0.001. Here, higher scores indicate a more favorable perception towards the assessment tool. The MCQ format was rated fairer (52%), whereas the essay format was rated fair by only 11% of students. Similarly, perceptions of learning varied widely across assessment formats, χ² [6] = 62; *p* < 0.001, with higher scores indicating greater perceived learning. OSCEs (60%) and MCQs (59%) were more commonly associated with enhanced learning compared to essays (25%). Participants’ preferences for how often each assessment method should be used also demonstrated significant differences, χ² [6] = 42; *p* < 0.001. Preference scores increased with more favorable responses (i.e., a preference for “using much more”). Essay questions (52%) and MCQs (48%) were most frequently indicated for increased use, whereas oral exams and portfolios were less frequently indicated for greater use (Table 1).

**Table 1:** Student perceptions of assessment tool formats employed in the harmonized curriculum

Parameters		Competency assessment tools						
		MCQ	Oral Exam	Essay Question	Portfolio	Seminar/journal presentation	Case (Morning/ Bedside) presentation	^a^OSCE
Difficulty	Difficult	2(2)	21(18)	59(50)	10(9)	33(28)	27(23)	39(33)
	Neutral	61(52)	80(68)	59(50)	40(34)	68(58)	52(44)	76(64)
	Easy	55(47)	17(14)	^*^	68(58)	17(14)	39(33)	3(2)
	Median (IQR)	2(2–3)	2(2–2)	1.5(1–2)	3(2–3)	2(1–2)	2(2–3)	2(1–2)
	Friedman Test: χ^2^(6) = 219.2; *p* < 0.001							
Fairness	Unfair	9(8)	24(20)	14(12)	19(16)	8(7)	9(8)	9(8)
	Neutral	48(41)	58(49)	91(77)	57(48)	55(47)	71(60)	58(49)
	Fair	61(52)	36(31)	13(11)	42(36)	55(47)	38(32)	51(43)
	Median (IQR)	3(2–3)	2(2–3)	2(2–2)	2(2–3)	2(2–3)	2(2–3)	2(2–3)
	Friedman Test: χ^2^(6) = 83.1; *p* < 0.001							
Degree of learning	Learn very little	*	23(19)	21(18)	21(18)	11(9)	3(2)	14(12)
	Neutral	49(42)	42(36)	67(57)	37(31)	51(43)	61(52)	33(28)
	Learn a lot	69(58)	53(45)	30(25)	60(51)	56(48)	54(46)	71(60)
	Median (IQR)	3(2–3)	2(2–3)	2(2–3)	3(2–3)	2(2–3)	2(2–3)	3(2–3)
	Friedman Test: χ^2^(6) = 62.1; *p* < 0.001							
Preferred frequency of use	Use much less	13(11)	21(18)	25(21)	23(19)	7(6)	12(10)	6(5)
	Neutral	48(41)	76(64)	32(27)	52(44)	69(58)	64(54)	57(48)
	Use much more	57(48)	21(18)	61(52)	43(36)	42(36)	42(36)	55(47)
	Median (IQR)	2(2–3)	2(2–2)	3(2–3)	2(2–3)	2(2–3)	2(2–3)	2(2–3)
	Friedman Test: χ^2^(6) = 42; *p* < 0.001							

a -Objective Structured Clinical Examination; *-No respondents rated. All values in parentheses represent percentages (%)

## Discussion

This study explored clinical pharmacy students’ views on different assessment methods used to evaluate their clinical skills. Overall, students appreciated formative assessments, recognizing their role in boosting motivation, academic success, and confidence. OSCEs were also viewed positively, with most students agreeing that they effectively measure knowledge, communication, and practical clinical skills. When comparing various assessment tools, MCQs and OSCEs were most often associated with better learning outcomes and were considered fair and useful for assessing competence. Conversely, essay and oral exams were seen as more challenging or less beneficial for learning. Students’ preferences for assessment methods varied widely, especially regarding perceived difficulty, fairness, educational value, and the frequency with which they should be conducted.

Students believed that formative assessment (FA) methods could help them identify their strengths and weaknesses in the subject, enabling them to pinpoint areas needing more attention. They also thought this approach could boost their academic performance, motivation, confidence, competency, fit within the program, and employability within the learning module. This might partly be due to the ongoing, systematic assessment process, which allows students to evaluate themselves regularly. Educators can quickly identify which aspects of the course and teaching require more focus and spot students who may need academic support. This aligns with existing research in healthcare professions education, which shows that FA encourages active learning and motivates students to engage more deeply with course content through ongoing feedback and reflection, helping students recognize their strengths and weaknesses, pinpoint specific areas for improvement, and assist instructors in promptly addressing students’ challenges [37–39]. Similarly, research by Van der Vleuten and colleagues [21] emphasizes that ongoing assessment and feedback are crucial elements of programmatic assessment, promoting competency growth over time. Additional studies show that students appreciate FA because it enables self-reflection and helps teachers identify learning gaps, leading to targeted academic support [40, 41]. Overall, these results imply that integrating structured formative assessment techniques into pharmacy education can enhance student learning, foster self-regulation, and facilitate the development of clinical skills.

In this study, many students reported using question-spotting as a key exam-preparation strategy, with some feeling overwhelmed by the large volume of study materials. They observed that their module grades often depended heavily on one or two high-stakes written exams and believed that continuous assessment was perceived as a fairer way to evaluate academic performance. Some students also found negative marking in MCQs unfair. These findings are consistent with previous research showing that students tend to adopt strategic learning approaches, like question-spotting, especially when assessment systems focus on high-stakes summative exams [40, 42]. Similar findings in health professions education suggest that students see continuous or multiple assessments as fairer and more supportive of learning than single end-of-term exams [41, 43]. Concerns about negative marking in MCQs have been raised earlier, indicating that such practices can increase test anxiety and discourage students from attempting questions, even with partial knowledge [44]. Overall, these findings emphasize the need for balanced assessment strategies that incorporate both formative and summative methods to provide a better assessment of student learning and clinical skills.

Despite the multiple advantages of the assessment approaches and their applicability in the curriculum, learners may not explicitly discuss how they perceive different assessment methods and the importance of distinguishing among them in real-world practice. Without a clear indication of which formats are used in formative versus summative settings, students may interpret al.l assessments as high-stakes, potentially affecting their learning behavior, stress levels, and overall perception of the educational experience. As outlined in the curriculum (pp. 11–12) [30], formats such as OSCEs, presentations, and logbooks may be employed in both formative and summative settings. However, the curriculum does not provide explicit contextual differentiation for these uses. This ambiguity might impact students’ views because certain formats are inherently summative, especially if they are graded or linked to progression decisions. Such experiences can influence student engagement, stress, and learning approaches. We believe that clearer communication of assessment intent (formative vs. summative) would enhance alignment between curriculum design and student perception. Clarifying this distinction within clinical practices would enhance the effectiveness of both the teaching and learning process.

Graduate students had positive attitudes towards the use of OSCEs to assess clinical competence. A larger proportion of students agreed and strongly agreed that the OSCEs could effectively evaluate their clinical understanding, integrate knowledge across modules, and assess the clinical skills required in pharmacy practice. A possible explanation is that if the OSCE is well integrated into existing assessment methods, it could validate and enhance the reliability of clinical assessment tools, thereby significantly increasing theoretical and practical knowledge. Students may therefore perceive OSCEs as accurate assessments of their skills. This view is strongly supported by earlier studies [45, 46], which describe the OSCE as a multipurpose clinical assessment tool employed to assess the clinical knowledge and skills of healthcare professionals in clinical settings. The OSCE, which evaluates competency in a precise, objective, and reproducible manner, allows uniform testing of students across an extensive range of clinical skills. It is particularly effective in assessing critical areas of healthcare professionals, such as communication skills and the ability to handle unpredictable patient behavior [45, 46].

More importantly, OSCEs are widely recognized as a reliable and structured instrument for assessing clinical competence, with a focus on evaluating the application and demonstration of clinical skills (“shows how”), which are essential elements of competency-based health professions education. As a result, it was highlighted as an essential assessment tool to better understand students’ perceptions of its effectiveness in evaluating clinical competencies [32, 47]. Furthermore, the OSCE has been increasingly utilized in both undergraduate and graduate programs worldwide. These assessment instruments are also employed in licensure examinations and as feedback instruments in FA approaches [47]. Therefore, the survey findings revealed that students have positive and engaging insights into the utilization of this instrument in evaluating their clinical learning outcomes and have positive predictions in utilizing it to measure clinical competencies.

This survey also explored students’ perceptions of the difficulty, fairness, and effectiveness of various assessment instruments, as well as their preferences regarding their clinical skill assessment process. Although a higher proportion of students did not express strong opinions or take a definitive stance, about half rated the essay-type questions as the most difficult tool, followed by the OSCE. Despite many students (68%) having agreed or strongly agreed with the utilization and objectivity of OSCE in assessing clinical competencies, they also rated it as difficult to perform each OSCE. This may be explained by the OSCE format, which integrates pharmacotherapeutic knowledge, problem-solving, and interpersonal skills, allowing them to learn from mistakes before real patient encounters [48]. This was supported by previous reports stating that, despite various difficulties, the OSCE has received considerable support from students, who felt it provided significant knowledge across all stations [33]. Conversely, portfolios and MCQs were considered the easiest assessment methods. These findings suggest how learners perceive different assessment methods and indicate areas where educators might need to address perceived difficulties. This can help in modifications and adjustments to better utilize these assessment tools in evaluating clinical competencies. Additionally, approximately 60% of students reported gaining significant knowledge of many of these tools, and those who did used them much more frequently.

However, this study has important implications for pharmacy education; the findings, with some limitations, warrant consideration, and the results should be interpreted carefully. This study was carried out at a single institution with a relatively small sample size, which may limit the generalizability of the results. The cross-sectional design and reliance on self-reported data prevent establishing causality and may introduce bias. Furthermore, the study did not evaluate actual learning outcomes or the effectiveness of assessment tools in real clinical settings. The potential influence of student attributes such as academic level, program type, or prior experience was not examined. Future longitudinal research should address these gaps and examine their impact on perceptions and assessment-related outcomes.

## Conclusion

This survey provided valuable insights into clerkship pharmacy students’ perceptions of assessment methods and the various assessment tools used in a competency-based curriculum. FA were seen to boost motivation, confidence, and ongoing engagement with learning, aligning with students’ preference for assessments that promote academic development rather than merely ranking performance. While SA played a key role in determining grades, which are often associated with stress and extensive preparation, it also highlights the need for effective strategies. The OSCE was regarded as an effective way to assess integrated clinical competencies and practical skills, although it was considered somewhat challenging to perform.

## Data Availability

The data and materials supporting the findings of this study are available from the corresponding author upon reasonable request.

## Abbreviations

AFL: Assessment for Learning
AOL: Assessment of Learning
CBE: Competency-Based Education
FA: Formative Assessments
MCQ: Multiple-choice Question
OSCE: Objective Structured Clinical Examination
SA: Summative Assessment

## References

[1] Nash RE, et al. An international review of the use of competency standards in undergraduate pharmacy education. Pharmacy Education. 2015;15(0). https://pharmacyeducation.fip.org/pharmacyeducation/article/view/324.

[2] Bruno A, et al. Towards a global competency framework. American Journal of Pharmaceutical Education. 2010;74(3):56. 10.5688/aj740356 PMC2865424 20498749

[3] Miller BM, Moore DE Jr, Stead WW, Balser JR. Beyond Flexner: a new model for continuous learning in the health professions. Acad Med. 2010;85(2):266–72. 20107354 10.1097/ACM.0b013e3181c859fb

[4] Atkinson J, De Paepe K, Pozo AS, Rekkas D, Volmer D, Hirvonen J, et al. The second round of the PHAR-QA survey of competences for pharmacy practice. Pharmacy. 2016;4(3):27. 28970400 10.3390/pharmacy4030027 PMC5419365

[5] Lockyer J, Carraccio C, Chan M-K, Hart D, Smee S, Touchie C, et al. Core principles of assessment in competency-based medical education. Med Teach. 2017;39(6):609–16. 28598746 10.1080/0142159X.2017.1315082

[6] Wolters M, van Paassen JG, Minjon L, Hempenius M, Blokzijl M-R, Blom L. Design of a Pharmacy Curriculum on Patient Centered Communication Skills. Pharmacy. 2021;9(1):22. 33467691 10.3390/pharmacy9010022 PMC7838998

[7] Al-Ghananeem AM, Malcom DR, Shammas S, Aburjai T. A Call to Action to Transform Pharmacy Education and Practice in the Arab World. Am J Pharm Educ. 2018;82(9):7014. 30559504 10.5688/ajpe7014 PMC6291664

[8] Koster A, Schalekamp T, Meijerman I. Implementation of competency-based pharmacy education (CBPE). Pharmacy. 2017;5(1):10. 10.3390/pharmacy5010010. 10.3390/pharmacy5010010 PMC5419394 28970422

[9] International Pharmaceutical Federation (FIP). Strategic plan 2019-2024. The Hague, International Pharmaceutical Federation, 2019.

[10] Miller GE. The assessment of clinical skills/competence/performance. Acad Med. 1990;65(9):S63–7. 2400509 10.1097/00001888-199009000-00045

[11] Medina MS, et al. Center for the Advancement of Pharmacy Education 2013 educational outcomes. American Journal of Pharmaceutical Education. 2013;77(8):162. 10.5688/ajpe778162 PMC3806946 24159203

[12] Webb DD, Lambrew CT. Evaluation of physician skills in cardiopulmonary resuscitation. J Am Coll Emerg Physicians. 1978;7(11):387–9. 10.1016/s0361-1124(78)80158-0 45678

[13] Alfaifi S, Arakawa N, Bridges S. The relevance of the International Pharmaceutical Federation Global Competency Framework in developing a country-level competency framework for pharmacists: A cross-sectional study. Exploratory Res Clin Social Pharm. 2022;5:100095. 10.1016/j.rcsop.2021.100095 PMC9030276 35478515

[14] Cate tenO. Entrustment as assessment: recognizing the ability, the right, and the duty to act. J Graduate Med Educ. 2016;8(2):261–2. 10.4300/JGME-D-16-00097.1 PMC4857524 27168900

[15] Pittenger AL, et al. Entrustable professional activities for pharmacy practice. American journal of pharmaceutical education. 2016;80(4):57. 10.5688/ajpe80457 PMC4891855 27293224

[16] Holmboe ES, Sherbino J, Long DM, Swing SR, Frank JR. The role of assessment in competency-based medical education. Med Teach. 2010;32(8):676–82. 20662580 10.3109/0142159X.2010.500704

[17] Anderson HM, Anaya G, Bird E, Moore DL. A review of educational assessment. Am J Pharm Educ. 2005;69(1–5):84.

[18] Harris P, Bhanji F, Topps M, Ross S, Lieberman S, Frank JR, et al. Evolving concepts of assessment in a competency-based world. Med Teach. 2017;39(6):603–8. 28598736 10.1080/0142159X.2017.1315071

[19] Alfadl AA. Chapter 11 - Assessment Methods and Tools for Pharmacy Education, in Pharmacy Education in the Twenty-First Century and Beyond, A.I. Fathelrahman, et al., Editors. Academic Press. 2018. p. 147-168.

[20] Murray E, Gruppen L, Catton P, Hays R, Woolliscroft JO. The accountability of clinical education: its definition and assessment. Med Educ. 2000;34(10):871–9. 11012938 10.1046/j.1365-2923.2000.00757.x

[21] Van der Vleuten CP, Schuwirth L, Driessen E, Dijkstra J, Tigelaar D, Baartman L, et al. A model for programmatic assessment fit for purpose. Med Teach. 2012;34(3):205–14. 22364452 10.3109/0142159X.2012.652239

[22] Jacob SA, Power A, Portlock J, Jebara T, Cunningham S, Boyter AC. Competency-based assessment of practice-based experiential learning in undergraduate pharmacy programmes. Pharm Pract. 2021;19(4):1–7. 10.18549/PharmPract.2021.4.2482 PMC9013191 35474652

[23] Van Der Vleuten CP. The assessment of professional competence: developments, research and practical implications. Adv Health Sci Educ. 1996;1(1):41–67. 10.1007/BF00596229 24178994

[24] Van Der Vleuten CP, Schuwirth LW. Assessing professional competence: from methods to programmes. Med Educ. 2005;39(3):309–17. 15733167 10.1111/j.1365-2929.2005.02094.x

[25] Black P, Wiliam D. Inside the Black Box: Raising Standards through Classroom Assessment. Phi Delta Kappan. 1998;80(2):139–48.

[26] Adams WK, Wieman CE. Development and validation of instruments to measure learning of expert-like thinking. Int J Sci Educ. 2011;33(9):1289–312.

[27] Stiggins RJ. Assessment crisis: The absence of assessment for learning. Phi Delta Kappan. 2002;83(10):758–65.

[28] Black P, Wiliam D. Assessment and Classroom Learning. Assessment in Education: Principles, Policy & Practice. 1998;5(1):7–4.

[29] Shepard LA. Linking formative assessment to scaffolding. Educational Leadersh. 2005;63(3):66–70.

[30] Education Mo. Nationally Harmonized Modular Curriculum for Bachelor’s Degree in Pharmacy (B.Pharm). Ethiopia: Addis Ababa; 2013. p.1-357.

[31] Dixson DD, Worrell FC. Formative and summative assessment in the classroom. Theory Into Pract. 2016;55(2):153–9.

[32] Witheridge A, Ferns G, Scott-Smith W. Revisiting Miller’s pyramid in medical education: the gap between traditional assessment and diagnostic reasoning. Int J Med Educ. 2019;10:191. 31655795 10.5116/ijme.5d9b.0c37 PMC7246123

[33] Awaisu A, Abd Rahman NS, Mohamed MHN, Bux SHBR, Nazar NIM. Malaysian pharmacy students’ assessment of an objective structured clinical examination (OSCE). Am J Pharm Educ. 2010;74(2):34. 20414449 10.5688/aj740234 PMC2856427

[34] Salinitri FD, O’Connell MB, Garwood CL, Lehr VT, Abdallah K. An objective structured clinical examination to assess problem-based learning. Am J Pharm Educ. 2012;76(3):44. 22544961 10.5688/ajpe76344 PMC3327242

[35] Crisp GT. Integrative assessment: Reframing assessment practice for current and future learning. Assess Evaluation High Educ. 2012;37(1):33–43.

[36] Dolin J, et al. Exploring Relations Between Formative and Summative Assessment, in Transforming Assessment: Through an Interplay Between Practice, Research and Policy, J. Dolin and R. Evans, Editors. Cham: Springer International Publishing; 2018;453–80.

[37] Black P, Wiliam D. Assessment and classroom learning. Assess Education: Principles Policy Pract. 1998;5(1):7–74.

[38] Gikandi JW, Morrow D, Davis NE. Online formative assessment in higher education: A review of the literature. Comput Educ. 2011;57(4):2333–51.

[39] Yorke M. Formative assessment in higher education: Moves towards theory and the enhancement of pedagogic practice. High Educ. 2003;45:477–501.

[40] Sambell K, McDowell L, Montgomery C. Assessment for Learning in Higher Education. 1st ed. Routledge. 2012.

[41] Carless D. Excellence in University Assessment: Learning from award-winning practice. ed. s, editor: Routledge. 2015.

[42] Svensäter G, Rohlin M. Assessment model blending formative and summative assessments using the SOLO taxonomy. Eur J Dent Educ. 2023;27(1):149–57. 35132742 10.1111/eje.12787 PMC10078662

[43] Nicol DJ, Macfarlane-Dick D. Formative assessment and self‐regulated learning: a model and seven principles of good feedback practice. Stud High Educ. 2006;31(2):199–218.

[44] Downing SM. The effects of violating standard item writing principles on tests and students: the consequences of using flawed test items on achievement examinations in medical education. Adv Health Sci Educ Theory Pract. 2005;10(2):133–43. 16078098 10.1007/s10459-004-4019-5

[45] Zayyan M. Objective structured clinical examination: the assessment of choice. Oman Med J. 2011;26(4):219. 22043423 10.5001/omj.2011.55 PMC3191703

[46] Hamann C, Volkan K, Fishman MB, Silvestri RC, Simon SR, Fletcher SW. How well do second-year students learn physical diagnosis? Observational study of an Objective Structured Clinical Examination (OSCE). BMC Med Educ. 2002;2:1–11. 11888484 10.1186/1472-6920-2-1 PMC80153

[47] Khan KZ, Ramachandran S, Gaunt K, Pushkar P. The objective structured clinical examination (OSCE): AMEE guide 81. Part I: an historical and theoretical perspective. Med Teach. 2013;35(9):e1437–46. 23968323 10.3109/0142159X.2013.818634

[48] Cerveny JD, Knapp R, DelSignore M, Carson DS, Bultemeier NC, editors. Experience with objective structured clinical examinations as a participant evaluation instrument in disease management certificate programs. Conference on Certificate Programs in Pharmacy in. 1998; Citeseer.

